# Evaluation of two easy-to-implement digital breathing interventions in the context of daily stress levels in a series of N-of-1 trials: results from the Anti-Stress Intervention Among Physicians (ASIP) study

**DOI:** 10.1038/s41746-025-02317-3

**Published:** 2026-01-10

**Authors:** Valentin Max Vetter, Tobias Kurth, Stefan Konigorski

**Affiliations:** 1 Institute of Public Health, Charité—Universitätsmedizin Berlin, Berlin, Germany; 2 Department of Endocrinology and Metabolic Diseases (including Division of Lipid Metabolism), Biology of Aging working group, Charité—Universitätsmedizin Berlin, Berlin, Germany; 3 Digital Health—Machine Learning Group, Hasso-Plattner-Institute for Digital Engineering, Potsdam, Germany; 4 Hasso Plattner Institute for Digital Health at Mount Sinai, Icahn School of Medicine at Mount Sinai, New York, NY USA; 5 Department of Statistics, Harvard University, Boston, MA USA; 6 Digital Engineering Faculty, University of Potsdam, Potsdam, Germany

**Keywords:** Clinical trial design, Research data, Patient education

## Abstract

Physicians face intense work-related stress, which can harm their health, increase the risk of medical errors, lower healthcare quality, and increase costs within the healthcare system. In this 4-week intervention study, individual-level and population-level effects of two short and easy-to-perform breathing exercises designed to reduce stress are evaluated among 76 physicians in residency in Germany in a series of N-of-1 trials. Levels of stress and levels of stress expected for the following day were assessed electronically every day via the StudyU App (protocol adherence: 91.9%). Average intervention effects were estimated using Bayesian linear regression models. They were overall small on the population level, but they showed large heterogeneity between individuals, with strong effects for selected individuals, with stress reduction of up to 3 points on a 1 to 10 stress scale. Thirty-one participants benefited from the anti-stress exercises. Three (mindfulness breathing) and seven participants (box breathing) had a ≥70% probability for a daily stress reduction of ≥0.5 points and thereby fulfilled our responder criteria. Of the 17 participants who completed the follow-up survey about 4.5 months after completion of the individual N-of-1 trials, 58% reported that they felt they had benefited from the intervention and 42% planned to use it in the future. The results highlight the value of personalized perspectives: while the studied interventions showed only small positive benefits for the “average person”, they may well help actual individual persons, here 10 of 76 or even 31 of 76 participants.

## Introduction

Physicians are regularly exposed to work-related and emotional stressors^1,2^, which, together with inadequate self-care^3^, are responsible for the frequently reported high stress burden in this group (reviewed in ref. ^2^). The rate of burnout and emotional distress among physicians was shown to be between 23 and 76%^4–11^, and 22% of German physicians in residency are reported to have taken medication because of work-related stress^12^. Among physicians, high work-related stress and burnout were associated with symptoms of depression^13^, increased clinically significant psychiatric morbidity^14^ and increased prevalence of suicidal thoughts^15^. Differences in burnout rates between specialties were found^7^, and the stress burden was higher in younger physicians and physicians in residency^2,5,16^.

Exposure to acute stress activates two main physiological systems. The sympatho-adrenal medullary (SAM) system releases catecholamines, which affect various physiological responses, including heart rate, blood pressure, and the pulmonary system, preparing the body for a “fight or flight” response when threatened^17,18^. The hypothalamo-pituitary adrenal (HPA) axis causes the release of glucocorticoids, which have anti-inflammatory effects, promote gluconeogenesis, and modulate various metabolic processes^18–20^. A frequent or prolonged activation of these systems increases the risk for diseases^21,22^ mostly through their immune-modulatory effects^23,24^. Additionally, high levels of stress are associated with unhealthy behaviors such as sleep deprivation and decreased exercising^19^, increased nicotine and alcohol consumption^25,26^, substance abuse^27^, and impulsive decision making^28^. The association of psychological stress, disease and other adverse health outcomes for the average individual experiencing stress is well-established^19,22,29–31^.

However, in physicians, high levels of stress and burnout were shown to have effects exceeding the average level as they were associated with increased risk for deviation from medical standards and major medical errors, poorer doctor-patient relationships, and overall lower quality in patient care^5,32–41^. Therefore, reducing stress levels among physicians would increase the physical and mental health of the medical workforce and could potentially improve patient care^42^. The reduction of stress in physicians is also of economic interest^43^. High levels of stress were shown to be associated with high turnover rates, burnout, early retirement, and lower productivity rates among physicians^38,44–47^, which in turn substantially increases costs within the healthcare system^2,43^.

For this reason, the Anti-Stress Intervention Among Physicians (ASIP) study evaluates two easy-to-learn breathing interventions that can be flexibly performed and do not require special training or additional tools, and are therefore easily incorporated into everyday routine. The first intervention, a guided 8-minute mindfulness and breathing exercise^48,49^, includes stretching and simple upper body movements. The second intervention, box breathing (also known as “tactical breathing”), was shown to decrease heart rate and is used by military and law enforcement to cope with stressful situations^50–52^. Beneficial effects of breathing exercises^49,53,54^ and mindfulness interventions^42,48,55,56^ on stress, anxiety, and well-being were shown in some studies, but other studies did not find a statistically discernible (i.e., statistically significant)^57–60^ effect of these interventions^48,54,61,62^. A large degree of heterogeneity of the individual-level intervention effects is expected^63^, as previous studies showed differences in effect size between study populations in dependence on the characteristics of their participants^64^. Furthermore, Holman and colleagues argue that the realization of beneficial effects of anti-stress interventions can depend on contextual factors such as motivation and initial levels of well-being, and stress the need for studies investigating the individual-level intervention effect^65^.

In this study, we assess the effect of two anti-stress interventions on daily stress levels as well as the daily level of stress expected for the following day in a large series of N-of-1 trials^66^. N-of-1 trials are a modern trial design with applications across all fields of medicine and the behavioral sciences, including the field of neurology and rare disease^67^, and they allow the estimation of individual-level effects, population-level effects, and between-participant differences in the individual-level intervention effects. This allows the researcher to report on intervention effects that were observable for different individuals, even if an effect on the group level was not observable^68^. Despite its well-established methodology and advantages in the context of individual-level effect estimation and implications for personalized medicine, it is rarely used in medical research. In our study, each participant went through a randomly allocated pre-specified 4-week sequence of intervention and control phases, and reported their stress level on each day. After trial completion, the individual-level intervention effects were assessed by comparing the daily participant-reported outcomes (PROs) between phases.

Separately for each of the two interventions, the population-level intervention effects were estimated by aggregating the data of the individual N-of-1 trials. This study design is considered to be the gold standard when dealing with strong effect heterogeneity^66^, and allows for individual feedback to each participant on the observed effects. An average of 4.5 months after completion of the individual N-of-1 trial, a longitudinal follow-up was conducted to assess the sustainability of the evaluated intervention effects. A carefully developed study design guaranteed full anonymity of all collected data while allowing horizontal cross-platform data linkage as well as longitudinal assessments.

In this study, we report on the individual-level and population-level effects of the two aforementioned anti-stress interventions, mindfulness breathing and box breathing, on the per-individual average daily level of stress and the per-individual average level of stress expected for the following day in 76 physicians in residency in Germany. We hypothesized that performing an anti-stress intervention reduces the per-individual average daily stress level and the per-individual average level of stress expected for the following day^69^ on average across individuals (i.e., population level) and within each individual. By providing a detailed previously published study protocol^69^, showcasing the use of modern trial software as well as making all data and statistical code openly available, we aim to promote the methodological benefits of this study design.

## Results

### Study participants

**Fig. 1:**
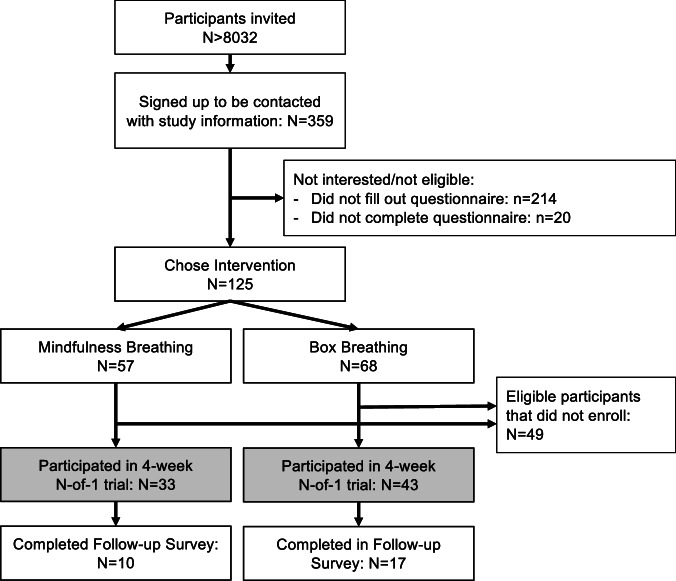
Chart of participants' flow in the ASIP study. After recruitment, participants were screened for eligibility and asked to fill out a baseline questionnaire, at which end they were asked to choose which intervention they would like to perform during their individual 4-week N-of-1 trial. On average, 4.5 months after their individual N-of-1 trial ended, participants were asked to fill out a follow-up survey.

Of the 8032 confirmed recipients of the study invitation, 359 participants indicated interest in participation and signed up to receive the study information and consent form. Of these participants, 125 completed the questionnaire. Thirty-three chose the mindfulness intervention (461 PROs documented over all trials), while 43 opted for the box breathing intervention (662 PROs documented over all trials, Fig. 1). Each N-of-1 trial consisted of a randomly allocated alternating sequence of intervention (A) and control (B) phases (ABAB or BABA), where each phase lasted 1 week for a total trial length of 4 weeks, and participants were asked to document the PROs daily. Participants were guided through their individual trial and reminded to perform the intervention by the StudyU App installed on their smartphone. Across interventions, 49.4% of all PROs were documented (i.e., had non-missing responses) during the intervention periods.

Participants were, on average, 35 years old, and 80% were women. Their overall self-rated health was good or very good (88%), and 65.8% of the participants reported drinking alcohol less than once per week. A large majority reported having never smoked (76.3%). Participating physicians in residency were, on average, in their fourth year of clinical training with, on average, 35 contractually agreed working hours per week (Table 1). Participants generally showed high values in the psychological questionnaires aimed at quantifying individual chronic stress, burnout symptoms, work-related privacy conflicts, over-commitment at work, and a low effort-reward ratio. Life satisfaction was in a range comparable to the general public (Table 1). Chronic stress levels, assessed using Cohen’s 10-item Perceived Stress Scale (PSS), were correlated with patient-reported outcomes (PROs) of stress assessed at baseline. Specifically, chronic stress showed a moderate correlation with daily stress levels (Pearson’s *r* = 0.5, 95% confidence interval: 0.3 to 0.6) and a weak correlation with anticipated stress levels for the following day (Pearson’s *r* = 0.2, confidence interval: 0.0 to 0.4). Lower average stress levels were documented during the weekend (Supplementary Figs. 1 and 2).

**Table 1:** Baseline data on participants were assessed via questionnaire prior to the start of the individual N-of-1 trials

	Mindfulness breathing (*n* = 33)	Box breathing (*n* = 43)
**Variables**	**Mean (SD),** ***n*** **(%)**	**Mean (SD),** ***n*** **(%)**
Age (years)	35.79 (6.3)	34.98 (6.27)^a^
Sex (female)	30 (90.9%)	30 (71.4%)^b^
Self-reported overall health		^a^
Very good	9 (27.3%)	10 (23.8%)
Good	22 (66.7%)	25 (59.5%)
Mediocre	2 (6.1%)	6 (14.3%)
Bad	0 (0.0%)	1 (2.4%)
Very bad	0 (0.0%)	0 (0.0%)
Sport (days/week)	1.94 (1.34)	2.28 (1.65)
Alcohol		
Once per week or more	8 (24.2%)	18 (41.9%)
Less than once per week	21 (63.6%)	21 (48.8%)
Not in the past 12 months or never	4 (12.1%)	4 (9.3%)
Smoking		
Yes, current	0 (0.0%)	1 (2.3%)
No, former smoker	6 (18.2%)	11 (25.6%)
No, never smoker	27 (81.8%)	31 (72.1%)
Year of residency	3.76 (1.77)	4.02 (2.5)
Work hours as per contract (h/week)	34.91 (6.93)	35.79 (6.18)
Average overtime (h/week)	3.79 (7.7)	3.51 (5.69)
Workplace		
Practice	14 (42.4%)	19 (44.2%)
Hospital	16 (48.5%)	23 (53.5%)
Other	3 (9.1%)	1 (2.3%)
Shift work (no)	26 (78.8%)	36 (83.7%)
Work-Privacy Conflict Scale	63.79 (26.81)	64.33 (20.87)
Copenhagen Burnout Inventory	56.57 (14.95)	53.09 (16.66)
Effort Reward Imbalance Questionnaire	0.78 (0.26)	0.8 (0.28)
Over-Commitment Scale	16.42 (3.51)	15.23 (2.6)
Cohen’s Perceived Stress Scale	20.52 (6.26)	19.02 (6.05)
Satisfaction With Life Scale	24.45 (5.82)	25.49 (5.91)

The mean and standard deviation are given for continuously scaled variables. The number of observations, as well as the percentage of the individuals in the respective category stratified by intervention groups, is stated for categorical variables.

*SD* Standard deviation, *n* number of observations, *h* hours.

^a^missing observations: *n* = 2

^b^missing observations: *n* = 1

### Individual-level analyses of each N-of-1 trial

Our primary outcomes were daily stress level and level of stress expected for the following day, both on a scale from 1 (“not stressed at all”) to 10 (“extremely stressed out”). The individual-level intervention effect can be interpreted as an average period treatment effect^70^ (i.e., average period intervention effect for each individual) or a conditional average treatment effect for the individual^71^ (i.e., average intervention effect conditional on that individual), comparing the mean stress level during the two intervention periods with the mean stress level during the two control periods. For each participant, each mean is taken over all time points. For both primary outcomes, a stress level reduction of at least 0.5 points was defined to be a clinically relevant effect before data assessment.

In a first step, the individual-level intervention effect for both primary outcomes was estimated separately for each participant using Bayesian linear regression models with an autoregressive error structure (AR1) modeling the dependency of observations between days. The individual-level intervention effect was estimated as the posterior mean of the intervention coefficient, along with its 95% credible interval. (A non-zero intervention coefficient represents a non-zero difference in the mean outcome between intervention and control participants.) For both primary outcomes, we also report the posterior probability of having a reduction of at least 0.5 points. For each outcome, a participant with a 70% or higher posterior probability of surpassing this threshold was classified as a responder (i.e., to the intervention).

**Fig. 2:**
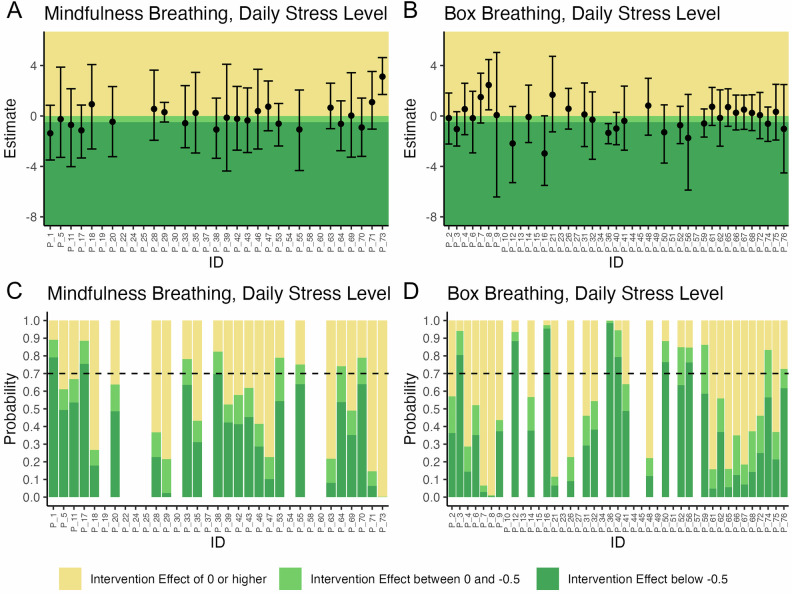
Results of the 76 individual N-of-1 trials (shown on the *x*-axis) with respect to daily stress levels. **A**, **B** Posterior means and 95% credible intervals of the difference in daily stress levels between intervention and control periods. **C**, **D** Posterior probabilities of any reduction of daily stress level (i.e., reduction > 0) and a clinically relevant stress reduction (i.e., reduction ≥ 0.5). If the posterior probability of stress reduction was 70% or higher, the participant was classified as a responder.

With respect to daily stress levels, substantial heterogeneity in the posterior mean of the individual-level intervention effect was observed. It ranged between −1.4 and 3.1 among participants who chose mindfulness breathing (Fig. 2A and Supplementary Table 1) and between −3.0 and 2.5 in the group that chose box breathing (Fig. 2B and Supplementary Table 2). In the group that performed mindfulness breathing, 14 participants had a negative posterior mean (i.e., showed decreased stress levels during the intervention phases) and 10 had a positive posterior mean (i.e., showed increased stress levels during the intervention phase), with all corresponding 95% credible intervals including zero. Of these, three were classified as responders, including one participant with a rounded posterior probability of 70% (i.e., 69.75%, Fig. 2C). Among participants performing box breathing, 17 participants had a negative posterior mean and (one of the 95% credible intervals excluded zero) and 15 participants had positive posterior mean (one of the 95% credible intervals excluded zero). Seven participants were identified as responders (Fig. 2D).

**Fig. 3:**
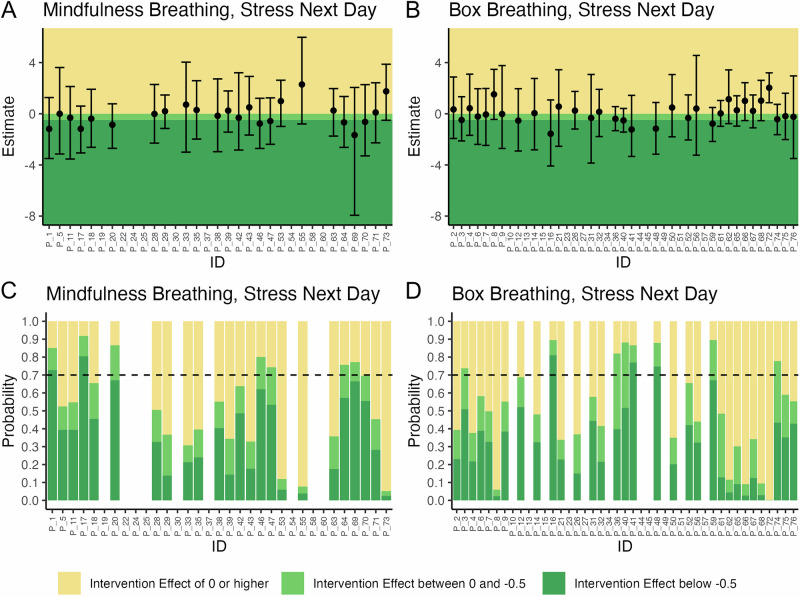
Results of the 76 individual N-of-1 trials with respect to levels of stress expected for the following day. **A**, **B** Posterior means and 95% credible interval of the difference in expected levels of stress of the next day between intervention and control periods. **C**, **D** Posterior probabilities of any reduction of expected level of stress on the next day (i.e., reduction > 0) and a clinically relevant stress reduction (i.e., reduction ≥ 0.5). If the posterior probability of a clinically relevant stress reduction was 70% or higher, the participant was classified as a responder.

Similarly, heterogeneous results were found for the expected stress levels on the next day, with posterior means ranging between −1.7 and 2.3 (mindfulness breathing, Fig. 3A and Supplementary Table 3) and −1.6 and 2.0 (Box breathing, Fig. 3B and Supplementary Table 4). Among participants performing mindfulness breathing, 13 had negative posterior means (with all 95% credible intervals including zero), and two were responders (Fig. 3C). Positive posterior means were observable in 10 participants (all corresponding 95% credible intervals included zero). Similarly, 16 of the study participants in the box breathing arm had negative posterior means below zero (with all 95% credible intervals including zero), and three were responders (Fig. 3D), while 16 had positive posterior means (one of the 95% credible intervals excluded zero).

The individual level time series data of reported stress levels are shown in Supplementary Figs. 1 and 2.

### Population-level analyses of each anti-stress intervention

The population-level intervention effect was an average treatment effect^70^ likewise comparing the mean stress level during the two intervention periods with the mean stress level during the two control periods. However, unlike the individual-level intervention effect, each mean is taken over all participants and time points.

Bayesian multilevel linear regression models with AR1 error structure were used to calculate posterior means, the corresponding 95% credible intervals, and the corresponding posterior probabilities of reaching a clinically relevant stress reduction. Outcomes were clustered by participant; i.e., participants were the highest hierarchical level of the models.

**Fig. 4:**
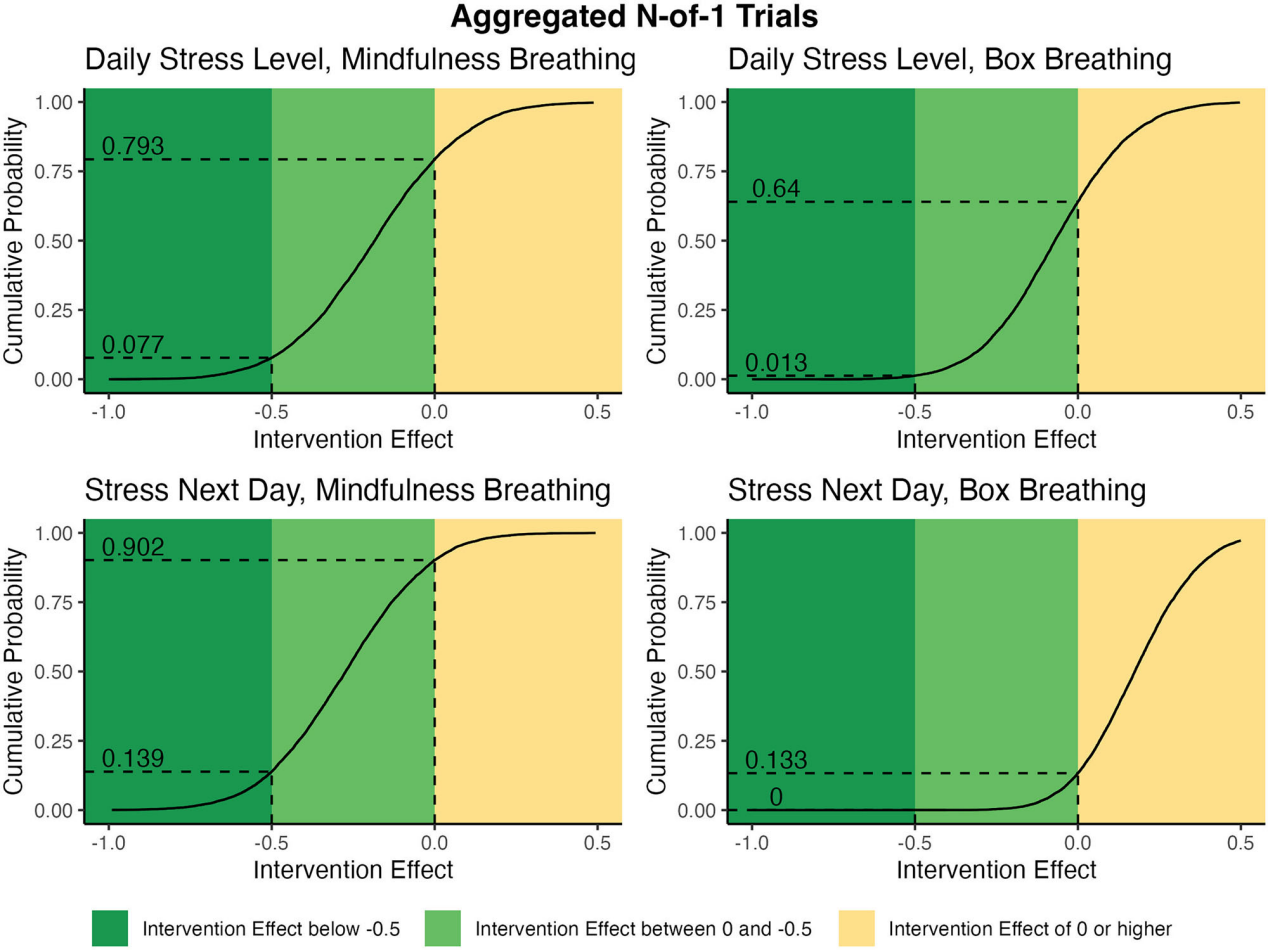
Cumulative posterior probabilities for the population-level intervention effect of the anti-stress interventions on the daily stress level and level of stress expected for the next day to be higher than a given value (on the *x*-axis). Posterior probabilities of any stress level reduction (i.e., reduction > 0) and a clinically relevant stress reduction (i.e., reduction ≥ 0.5) are marked with dotted lines.

On average, among participants who chose to perform mindfulness breathing, the intervention reduced the daily stress level by 0.18 points (95% credible interval: −0.62 to 0.26). The posterior probability of reducing the daily stress level at all by performing the intervention (i.e., a reduction of more than 0 points) was 79.3%. The posterior probability of a clinically relevant stress reduction of at least 0.5 points was 7.7% (Fig. 4). On average, among participants doing box breathing, the intervention reduced the daily stress level by 0.07 points (95% credible interval: −0.44 to 0.32). The posterior probability of reducing the daily stress level at all by performing box breathing was 64.0%, and that of a clinically relevant stress reduction was 1.3% (Table 2 and Fig. 4).

**Table 2:** Table of mean (M), standard deviation (SD), number of documented PROs (*n*), and mean difference in outcome variables between intervention and control periods (Dif.) in 76 participants of the ASIP study stratified by intervention group and primary outcome

	Intervention			Control			Dif.	Estm.	L 95% CI	Up 95% CI	p. clin. rel.
	M	SD	n	M	SD	n					
Daily stress											
Mindfulness breathing	3.95	2.49	215	4.09	2.59	246	−0.14	−0.18	−0.62	0.26	0.0774
Box breathing	3.92	2.24	340	4.01	2.07	322	−0.09	−0.07	−0.44	0.32	0.0128
Next day											
Mindfulness breathing	4.08	2.7	215	4.29	2.63	246	−0.21	−0.27	−0.69	0.14	0.1387
Box breathing	4.43	2.27	340	4.34	2.13	322	0.09	0.18	−0.13	0.51	0.0001

Bayesian linear regression models were used to calculate the posterior mean (Estm.) and 95% credible interval (CI) as well as the estimated average probability of a clinically relevant stress reduction (i.e., 0.5 points or more).

*M* Mean, *SD* Standard Deviation, *n* number of observations, *Dif.* Difference between average stress levels in intervention and control period, *Estm.* mean of posterior distribution in the Bayesian multilevel model of the aggregate data, *L CI 95%* lower 95% credible interval, *Up CI 95%* upper 95% credible interval, *p. clin. rel.* probability of a clinically relevant stress reduction.

For the level of stress expected for the next day, mindfulness breathing reduced the expected stress level by an average of 0.27 points (95% credible range: −0.69 to 0.14). The probability of reducing the expected stress level at all was 90.2%, and that of a clinically relevant stress reduction was 13.9%. Among participants using box breathing, the intervention increased the expected stress level by an average of 0.18 points (95% credible interval: −0.13 to 0.51). The probability of a clinically relevant stress reduction was <0.01% (Table 2).

### Secondary analyses: effect modifiers and population-level intervention effect of intervention-availability across study arms

To exploratively analyze potential effect modifiers, post hoc subgroup analyses stratified by sex and chronic stress level at baseline were performed. Among women, we estimated mean effects (and effect variability) similar to those observed over all participants. However, the estimated population-level stress reduction from mindfulness breathing was notably lower for both daily stress and expected stress.

Among men, mindfulness breathing reduced the daily stress level an average of 1.25 points (95% credible interval: −4.32 to 2.40), with a 75% posterior probability of a clinically relevant stress reduction. Similarly, performing this intervention reduced the level of stress expected for the following day on average by 0.57 points (95% credible interval: −6.77 to 3.39), and the posterior probability of a clinically relevant stress reduction was 48.4% among men. Interestingly, box breathing was estimated to result, on average, in an increase of daily stress among male participants (mean posterior: 0.1, 95% credible interval: −0.69 to 0.95) as well as of the level of stress expected for the next day (mean posterior: 0.24, 95% credible interval: −0.44 to 0.96, Supplementary Table 5).

Among participants with low chronic stress at baseline (<50th percentile of Cohen’s PSS), mindfulness breathing did not lead to a reduction in daily stress levels (posterior mean: −0.04, 95% credible interval: −0.73 to 0.65) or stress levels expected for the next day (posterior mean: −0.05, 95% credible interval: −0.67 to 0.58). However, a reduction of 0.32 points in the stress level on each day (95% credible interval: −0.97 to 0.31) was observable among participants performing box breathing and 0.17 points in the level of stress expected for the following day (95% credible interval: −0.65 to 0.36).

Among participants with high levels of chronic stress, lower daily stress levels (posterior mean: −0.25, 95% credible interval: −0.92 to 0.44), as well as lower levels of expected stress (posterior mean: −0.29, 95% credible interval: −0.96 to 0.38), were found among participants evaluating mindfulness breathing during the intervention periods. However, among participants evaluating box breathing, higher stress levels in this group (daily stress: posterior mean: 0.13, 95% credible interval: −0.35 to 0.64; expected stress next day: posterior mean: 0.38, 95% credible interval: −0.06 to 0.83, Supplementary Table 6) were documented after performing the intervention.

Finally, by aggregating both groups, we estimated the average effect of performing an anti-stress intervention among participants using the intervention of their choice. No population-level intervention effect was observable when analyzing the aggregated data from both study arms together (Supplementary Table 7).

### Follow-up

A follow-up questionnaire was sent to all participants on average 4.5 months after the individual N-of-1 trials ended. Successful matching of a completed follow-up questionnaire with baseline data was possible for 10 (mindfulness breathing) and 17 participants (box breathing). Across interventions, 58% of participants felt that they benefited from performing the intervention, and 42% planned to use it in the future. Box breathing was recommended to others by 47.1%. However, only 30% of participants who evaluated mindfulness breathing recommended the intervention to others. While 40% of the participants from the mindfulness breathing group performed the intervention after completion of the N-of-1 trial, more than 82% of participants from the box breathing group reported using the intervention in their daily life (Table 3). Of all participants who filled out the follow-up questionnaire, one reported trouble breathing and dizziness after performing box breathing.

**Table 3:** Descriptive statistics of the response collected as part of the follow-up questionnaire, which was sent out to all participants on average 4.5 months after completion of the individual N-of-1 trials

	Mindfulness breathing	Box breathing
	*n*(%)	*n*(%)
The intervention has reduced my stress level.		
Disagree	0 (0.0)	1 (6.2)
Somewhat disagree	4 (40.0)	6 (37.5)
Somewhat agree	6 (60.0)	8 (50.0)
Agree	0 (0.0)	1 (6.2)
I plan to use the intervention allocated to me in the future.		
Disagree	1 (10.0)	1 (6.2)
Somewhat disagree	6 (60.0)	7 (43.8)
Somewhat agree	3 (30.0)	5 (31.2)
Agree	0 (0.0)	3 (18.8)
Have you recommended the intervention to someone else?		
No	7 (70.0)	9 (52.9)
Yes, to a colleague	2 (20.0)	2 (11.8)
Yes, to a friend	1 (10.0)	1 (5.9)
Yes, to my family	0 (0.0)	1 (5.9)
Yes, to the patient	0 (0.0)	4 (23.5)
Do you use the intervention in your everyday life?		
Yes, daily or almost daily	0 (0.0)	1 (5.9)
Yes, on 1–6 days per week	1 (10.0)	4 (23.5)
Yes, less than once per week	0 (0.0)	0 (0.0)
Yes, once a month or less	3 (30.0)	9 (52.9)
No, never	6 (60.0)	3 (17.6)

*n* number of observations.

## Discussion

In this study, we evaluated the effect of two short anti-stress interventions on the daily stress level and on the stress level expected for the following day in 76 physicians in residency in Germany. Due to an expected high heterogeneity in individual-level intervention effects (i.e., between individuals), a series of N-of-1 trials was conducted to estimate the individual-level and the population-level effect separately for each intervention using Bayesian linear regression models. Large between-individual differences were observable for both anti-stress interventions, with posterior means ranging between −3.0 and 3.1. A total of 31 participants experienced benefits from performing one of the anti-stress exercises. This effectiveness on the individual level was only observable due to the use of the innovative N-of-1 trial study design. This study design allowed to show that the intervention can indeed help a substantial number of participants, which would not have been identified in traditional population-level studies. To further examine differences across the individual-level intervention effects, sex- and stress-stratified subgroup analyses were conducted in an aggregate population-level analysis.

On the population level, a small directional reduction of the daily stress level was found for both mindfulness breathing (mean of posterior distribution of the treatment effect parameter in the Bayesian linear regression: −0.18) and box breathing (mean posterior estimate: −0.07), a trend that is in line with our hypothesis. While mindfulness breathing also reduced the expected stress levels for the following day (mean posterior estimate: −0.27), participants in the box breathing group, contrary to expectations, exhibited a slight increase in stress after performing the intervention (mean posterior estimate: 0.18). However, note that there was only weak inferential evidence for these estimated effects (i.e., 95% credible intervals all included zero).

Our exploratory secondary analyses observed differences in the population-level intervention effects between sex- and stress-stratified subgroups. In men, participants performing mindfulness breathing showed reduced stress levels, while among those performing box breathing, an increase in the perceived level of stress was observable. In the subgroup of women, no substantial differences to the results from the complete dataset were found. Participants with low levels of chronic stress benefited from box breathing, but no effect of mindfulness breathing was found. Among participants with high levels of chronic stress who performed mindfulness breathing, a reduction of stress levels during the intervention periods was documented. These results are in line with previous findings, which also reported stronger effects in participants with high baseline stress^60^. However, in highly stressed participants of the box breathing group, a slight elevation of stress perception was observed during the intervention phases compared to the control phases. Also note that there was only weak inferential evidence for these estimated effects. Further studies are needed to investigate the difference in intervention effects between sex and stress-level defined subgroups and other anti-stress interventions, for example, as planned in the study by Goodwin and colleagues^63^.

The large heterogeneity of the individual-level intervention effects suggests that the performance of breathing interventions can be highly effective for selected individuals but might not lead to a strong average stress reduction in larger groups. This was also observable in the exploratory subgroup analyses, which showed the largest population-level intervention effects for mindfulness breathing among men and participants with high baseline stress. The evident heterogeneity of intervention effects in this study adds to a growing body of literature that discusses the need for individualized and personalized medicine in contrast to a “one-size-fits-all” solution^72,73^. By informing decisions based on individual variables to optimize the treatment success, the effectiveness of the intervention can be optimized^74^. For example, the development of externally validated accurate prediction models on intervention success could inform individuals on their individual odds of experiencing beneficial results after performing the intervention. Previous studies demonstrated the benefits of this study design in the development of personalized treatment strategies^75,76^. As shown by the results of this study, N-of-1 trials can provide such information in a robust and transparent way and were mentioned to provide an ideal methodological framework in the context of personalized medicine^73,77^. N-of-1 trials are straightforward applicable to investigate digital interventions with immediate effects (while requiring more complex study designs and statistical methodology in case of interventions with lasting effects). N-of-1 trials are highly flexible, cost-effective and comfortable to use for study participants as they can choose their own time of enrollment in their own N-of-1 trial. Digital health interventions are particularly suitable to be used as they can be easily integrated into N-of-1 trial apps such as StudyU, and linked with digital assessment of health outcomes (either PROs or through wearables). Studies investigating other low-risk digital interventions in other fields have been successfully carried out with this methodological approach in the past, proving the reliability and applicability of this method (e.g., refs. ^78–80^). Alternatively, the N-of-1 trial approach allows individual feedback to each study participant on the efficacy of the intervention. This allows the participant to make an informed decision on whether they want to continue with the intervention after completion of their trial. This approach can be generalized to provide benefit in clinical or pharmacological settings where an individual and systematic assessment of the efficacy of a novel therapy could allow patients to make highly informed decisions based on their individually collected data.

The observed overall reduction in daily stress levels in this study due to the performance of anti-stress exercises is in line with previously published intervention studies conducted among physicians^81–84^. However, the population-level effect sizes found in this study are smaller compared to previous results. This could potentially be due to the short overall study duration, shorter and less intensive anti-stress interventions, and differences in the analyzed study population^81–84^. Smaller effects of shorter interventions compared to longer interventions or structured programs (meta-analyzed in ref. ^85^) were reported before, and a dose-response relationship between intervention length and stress reduction was suggested^86^. In this study, we aimed to compensate for the short duration and low dose of the interventions by instructing participants to perform the anti-stress intervention daily, which has been noted to increase the effect of anti-stress interventions^86^.

As expected (reviewed in ref. ^2^), our population sample showed a higher stress burden compared to the German general population. Specifically, we assessed work-related stress and conflict with private life (Work-Privacy Conflict Scale, WPCL) and personal burnout and exhaustion (Copenhagen Burnout Inventory, CBI) with two instruments from the Copenhagen Psychosocial Questionnaire (COPSOQ^87^). Compared to a German reference population (*n* = 2561^88^), the participants showed higher values in both scales (WPCL: ASIP mean: 64 vs. reference population mean: 45^88^; CBI: ASIP mean 55 vs. reference population 42^88^). Similarly, on the Over-Commitment Scale^89^ participants of the ASIP study showed, on average, higher values (mean: 16) compared to a sample of 10,698 people from the German general public that were assessed as part of the German Socio-Economic Panel (SOEP) (mean: 13)^89^. Compared to a randomly selected German community sample (*n* = 2463), ASIP participants showed elevated chronic stress levels assessed by Cohen’s PSS (ASIP: 20 vs. reference sample: 13)^90^. Interestingly, the perceived work-related stress measured by the Effort Reward Imbalance quotient (ERI questionnaire^89^) was slightly lower (mean: 0.79) compared to a sample of *n* = 3848 Germans (mean values between 0.83 and 0.89)^91^. The participants' life satisfaction was assessed with the Satisfaction With Life Scale (SWLS). It was at the same level as reported for the German validation sample for the respective questionnaire (mean in both studies: 25)^92^.

There are a number of limitations to this study. Firstly, the interventions were designed to be self-administrable and to be performed at the time of the participants' convenience. Therefore, differences in the time between intervention and documentation of PROs, as well as the time of day at which the intervention is performed, are expected. Secondly, as mentioned earlier regarding the 95% credible intervals, a large variance in the documented daily levels of stress within individuals, as well as between participants, was found, which might limit our ability to detect (i.e., infer) both individual-level and population-level intervention effects. In future studies, a daily assessment of systematic confounders could help to reduce the variance in the outcome measures. Thirdly, since the interventions were self-administered, we relied on participants’ self-reports to assess protocol adherence and were unable to electronically verify how the interventions were actually performed. However, the breathing exercises were accessible in the StudyU app only during the intervention phases and only prior to participants completing their daily PROs. As no alternative access to the interventions was possible, we sought to minimize the risk of participants performing the exercises outside the intended context. Fourthly, our study population might present a highly selected group of physicians in training, which may limit the generalizability of our findings to the broader target population. Given the response rate of about 1%, we expect that our participants were, on average, more motivated than our general target physician population concerning engaging in stress-reducing measures.

Strengths of this study include the N-of-1 study design, which allows for the estimation of individual- as well as population-level effects for each type of intervention. The large effect heterogeneity found in our study underlines the importance of using study designs that are able to analyze individual-level effects as well as between-person differences when evaluating anti-stress interventions. To our knowledge, this study is the first to investigate and test digital anti-stress breathing interventions using this methodological approach. Another strength of the study is its large sample size of 76 participants, which is more than twice the number of participants required (*n* = 34) according to our sample size calculation^69^. Previous results suggest that inconvenient scheduling and time constraints are the biggest hindering factors for physicians to use stress-reducing measures^93^. By exploring two brief, easy-to-use breathing exercises, we aim to address these challenges and promote a low-threshold integration into daily routines.

In summary, performing mindfulness breathing and box breathing results on average in a small reduction of daily stress levels. Large differences in the individual-level intervention effect were shown between participants and in secondary subgroup analyses. The most notable reduction in daily stress was observed among men and participants with high levels of chronic stress at baseline who practiced mindfulness breathing. Among participants evaluating box breathing, a reduction in stress levels during the intervention periods was observed for participants with low baseline stress, but a small increase in stress levels among men or participants with high baseline stress was found compared to control periods. Therefore, the results highlight the value of personalized perspectives: while mindfulness breathing and box breathing showed only small positive benefits for the “average physicians in residency”, some physicians in residency benefited substantially, while others exhibited an increase in perceived stress levels. This highlights the potential of digital N-of-1 trials and the protocol described here: interested individuals may wish to try these breathing exercises, evaluate their effectiveness, and continue them if they help individually or stop using them if they experience negative side effects, which are possible too. Additionally, our findings emphasize the importance of using study designs able to investigate effect heterogeneity when analyzing anti-stress interventions.

## Methods

### Trial design

The ASIP Study aims at the evaluation of two anti-stress interventions among healthy physicians in training in Germany. After screening for eligibility, participants were asked to fill out a baseline questionnaire and indicate their preference for mindfulness breathing or box breathing. The control condition was everyday life. Subsequently, participants were referred to the StudyU App^94^ and provided with an individual randomized sequence of two pairs of one-week intervention (A) and control (B) periods, resulting in a total trial duration of 4-weeks and two possible intervention-control-sequences (ABAB or BABA). In the StudyU App, participants were electronically guided through the 4-week study period and were provided with the digital anti-stress interventions. Three months after completing the collection of data from the individual N-of-1 trials, a follow-up questionnaire was sent out to all participants via email. The manuscript was prepared in accordance with the “CONSORT extension for reporting N-of-1 trials (CENT)”^95^. The published study protocol of this study can be accessed at ref. ^69^.

### Participants

The study was conducted in Germany among healthy physicians in training. Inclusion criteria were weekly working time in a clinical position of at least 9 h, regular access to a smartphone on which the StudyU App can be installed, and informed consent. Participants were excluded if they were <18 years of age, had already completed their specialist training, had no clinical activity during the study period (e.g., vacation, research activity, etc.), participated in another intervention study during the study period, did not speak German, performed yoga more than 4 times a month, meditated or performed breathing exercises on average more than 4 days per month, were confirmed or suspected to be pregnant or had a psychiatric disorder, cardiovascular disease, respiratory, pulmonary or neurological disease. Furthermore, participants were excluded from the study if they had a planned surgery within the next 6 months, reported substance abuse (for example, alcohol, drugs, or other), or a physician recommended (or self-assessment) not to perform mindfulness or breathing exercises. Due to data protection reasons, employees of the Charité—Universitätsmedizin Berlin were not eligible for study participation.

Recruitment of participants took place between April 15, 2024, and May 31, 2024, and was done via email, posting of study invitations in local messenger groups and on websites of institutes of university hospitals, as well as displaying the study invitation on seminar days for physicians in training. Interested participants were asked to provide their email addresses through a contact form and were subsequently sent the study information, the consent form, and a link to the baseline questionnaire. Additionally, participants were provided with contact information (email and phone number) of the study PI to discuss questions and obtain further information.

All participants provided informed consent. The study was conducted in accordance with the Declaration of Helsinki and approved by the Ethics Committee of the Charite—Universitätsmedizin Berlin - approval number EA4/260/23. The trial is registered at ClinicalTrials.gov under trial ID NCT06368791 (first registered April 11, 2024). No changes were made to the originally published study protocol^69^.

### Procedure

Data collection took place between April 15, 2024, and June 30, 2024. The carefully developed study design guaranteed anonymity of all collected data while maintaining cross-sectional and longitudinal data linkage across platforms used for data collection. Before beginning with the individual N-of-1 trials, participants were asked to fill out a baseline questionnaire, at the end of which they were presented with both anti-stress interventions and asked to choose which intervention they wanted to do as part of the study. This approach was intended to reflect a real-world scenario in which individuals can choose from a wide range of stress-reducing measures. Participants then received an individual linkage code to get access to the study in the StudyU App^94^. This mobile app for both iOS and Android phones was used to guide each participant through their individual trial, deliver the anti-stress interventions, and document the daily PROs. Participants were informed about their individual intervention-control sequence and were reminded by push notifications to perform the intervention (during intervention periods) and to document the PROs (during intervention and control periods). Anonymous data collected through the REDCap questionnaire and the StudyU App is publicly available (see below). Furthermore, the setup of the ASIP study, including the detailed N-of-1 procedure, as well as the respective interventions, is made publicly available for full transparency and easy replication within the StudyU App in the StudyU Designer (https://designer.studyu.health).

### Interventions

In this trial, two anti-stress interventions were tested independently. The first, mindfulness breathing, consisted of an 8-min guided mindfulness and breathing exercise. This intervention was delivered as an audio file through the StudyU App. Participants were asked to find a comfortable seated position in a quiet and low irritation environment. They were then instructed to perform a number of breathing exercises alongside simple body movements and stretching exercises. During the intervention, participants were advised to pay attention to conscious breathing, follow the flow of air through their body and focus on the feeling of calmness as a result of their natural breathing cycle. The second intervention, box breathing, was a 6-min structured breathing exercise presented as a video file. This video was seamlessly integrated and played within the StudyU App. Participants were instructed to breathe in for four seconds, hold their breath for four seconds, and breathe out for four seconds. After holding their breath for a further four seconds, the next breathing cycle began with four seconds of breathing in. Participants were provided with a visual aid in the form of a red dot following the outline of a blue square, which moved synchronously to the audio instructions and increased/decreased in diameter during the inspiration/expiration phases. Participants were instructed to perform the intervention only during the intervention phase of their N-of-1 trial. To reflect a real-world scenario, they were free to choose the timing of the intervention, as long as it was completed before their daily documentation of PROs.

### Control condition

Everyday life was used as a control for the intervention periods in which the participants performed an anti-stress intervention. Participants were specifically instructed not to perform the anti-stress intervention during this time. PROs were documented in the same way as during the intervention period.

### Outcomes

Two primary outcomes were documented on every day by the participants within the StudyU App:

- “Overall, how stressful was your day?”
- “Which level of stress do you expect for tomorrow?”

Each item was answered on a visual analog scale from 1 (“not stressed at all”) to 10 (“extremely stressed out”). Additionally, participants were asked whether they performed the anti-stress exercise on a given day. All three items were available to be answered by the participants after 4 pm on every day, and participants were reminded to document their PRO by push notification to their mobile phone.

### Effect modifiers and additional variables at baseline

At baseline, participants were asked to fill out an electronic baseline questionnaire hosted on REDCap (Research Electronic Data Capture)^96^. As part of this questionnaire, information on the participants’ demographics (e.g., age, sex, state of residence, relationship status, children in household), work (e.g., place of work, work hours per contract, average overtime), and lifestyle (e.g., alcohol, smoking, exercise) was collected.

Additionally, validated German versions of established psychological questionnaires were assessed which included the Work-Privacy Conflict Scale (Copenhagen Psychosocial Questionnaire, Cronbach’s alpha = 0.90)^87,88^, Copenhagen Burnout Inventory (Personal Burnout, Cronbach’s alpha = 0.84)^88^, Effort-Reward Imbalance Questionnaire (ERI, Cronbach’s alpha = 0.75)^89,97^, Over-Commitment Scale (OC, part of the ERI questionnaire, Cronbach’s alpha = 0.73)^89,97^, Cohen’s PSS (Cronbach’s alpha = 0.85)^98^, SWLS (Cronbach’s alpha = 0.90)^92^.

Following the procedure suggested by Nübling and colleagues^88^, participants who reported on less than 50% of the items in WPCS and the CBI were excluded from the analyses, and missing values were imputed by row-wise mean imputation. The ERI was only calculated for participants who did not have more than one missing item, as suggested by Beschoner and colleagues^99^, and missing values were imputed by the row-wise mean. In line with the literature^100^, this was done separately for the effort- and the reward-related items. The OC Scale was only calculated if two or fewer items were not answered, and missing values were imputed by row-wise mean. Cohen’s PSS and the SWLS were calculated if not more than one item was missing, and row-wise mean imputation was performed.

### Randomization

An alternating sequence of intervention (A) and control (B) periods was randomly assigned to each participant. Each sequence contained two pairs of alternating intervention and control periods, resulting in two possible sequences: ABAB and BABA. Participants were automatically and at random assigned to an individual linkage code at the end of the baseline questionnaire. In the StudyU App, each linkage code was filed before study start and alternately assigned to one of the two available randomization sequences (ABAB or BABA). Therefore, the researcher was blinded to the randomization as it was determined by the order in which the participants clicked on the invitation link.

#### Protocol adherence

For technical reasons, it was not possible to automatically document whether and for how long the intervention was carried out in the StudyU app. However, participants were asked daily during both the intervention and control phases whether they had completed the breathing exercise. Protocol adherence was high, with 91.9% of all documented PROs aligning with the pre-specified protocol. Specifically, among participants evaluating mindfulness breathing, 244/246 = 99.2% (control phase) and 176/215 = 81.7% (intervention phase) of PROs were documented following correct protocol adherence. Among participants performing box breathing, 259/286 = 91.0% (control phase) and 317/340 = 93.2% (intervention phase) of all PROs aligned with the respective protocol phase.

#### Follow-up

On October 1st and 2nd, 2024, 3 months after completing the collection of data from the individual N-of-1 trials, an email with an invitation to fill out a follow-up questionnaire was sent out to all participants. On average, the follow-up questionnaire was filled out 18.0 weeks after completing the N-of-1 trial, and the individual follow-up time ranged between 13.9 and 20.7 weeks. This outcome was anticipated, as the data for the N-of-1 trials were collected over a 2.5-month period, but all invitations to participate in the follow-up questionnaire were sent on the same date because, for anonymity reasons, the exact completion date of each individual trial was not known. Furthermore, the time between receiving the invitation and filling out the follow-up questionnaire differed between participants, which added variance in the follow-up time.

Longitudinal data linkage was done using a seven-digit Self-Generated Identification Code (SGIC) derived from specifically designed questions at the beginning of the baseline and follow-up questionnaire:

- Please enter the first and second letter of your mother’s first name.
- Please enter the first and second letter of your place of birth.
- Please indicate your sex as stated in your birth certificate.
- Please enter the number of your older (not younger!) siblings.
- Please enter the last digit of your parent’s house number.
- Please enter the last digit of your parent’s postal code.

Overall, 39 participants filled out the follow-up questionnaire. Exact matching was possible for 27 cases (69.2%). Fuzzy matching allowing a one-digit difference between SGICs using R’s stringdist_join function (fuzzyjoin package) resulted in four additional matches which were subsequently confirmed using demographic information provided at both time points. Therefore, successful longitudinal matching was possible in 31/39 = 79.5% of all cases. Of the matched cases, one (mindfulness breathing) and two participants (box breathing) filled out baseline and follow-up questionnaires but did not participate in their N-of-1 trial and were therefore excluded from the analyses. Additionally, one participant from the box breathing group filled out the follow-up questionnaire but did not provide answers to the questions reported in Table 3. To assess the usability of the StudyU-App, participants were asked to fill out the Systems Usability Scale (SUS, Cronbach’s alpha = 0.85) as part of the follow-up questionnaire^101^. Of all 27 participants who participated in the follow-up survey, 25 filled out the SUS, and all items were answered (no missing values). According to the interpretation proposed by Bangor and colleagues^102^, the average answer across all participants indicates an overall good usability of the StudyU-App (mean = 76.2, SD = 13.6). Individual answers ranged from 52.5 (corresponding to “OK”) to 95 (corresponding to “Best Imaginable”).

### Data management

All data was collected anonymously, i.e., no connection of any of the collected data with identifying information was possible at any point in time. Data were collected electronically using two platforms. The electronic baseline and follow-up surveys were conducted using REDCap^96^. The anonymous data collected as part of the questionnaire is stored on a protected server at the Charité— Universitätsmedizin Berlin, Berlin, Germany. Daily PROs were assessed through the StudyU App, a mobile application installed on each participant’s smartphone^94^. The data collected through the StudyU App is stored on a secure backend host on servers located at the Hasso Plattner Institute in Potsdam, Germany. Data collected from the baseline questionnaire and data recorded with the StudyU App were linked using the individual linkage code given to each participant at the end of the baseline questionnaire, and needed to access their N-of-1 trial in the StudyU App.

### Sample size

The sample size was calculated using the approach described by Yang and colleagues^103^. Based on results from the literature^104–106^, we assumed a homogenous residual standard error of 2.41, a within-individual autocorrelation of PRO responses of 0.8, and a reduction of the daily stress level by a standardized mean difference of at least 0.3. The level of statistical significance was set at *α* = 0.05. A statistical power of 80% was reached when at least six participants per sequence reported at least four PRO responses per 1-week phase during a 4-week trial period (per intervention: *n* = 2 × 6 = 12). To account for a 30% dropout rate, *n* = 34 participants were determined to be the required sample size for recruitment^69^.

### Statistical methods

Descriptive statistics of the study population are presented as mean, standard deviation, minimum, and maximum or number of observations and percentages in Table 1.

Bayesian multilevel models employing the Markov Chain Monte Carlo method are used to estimate the posterior distribution of the average effect of each of the two investigated interventions on the two primary outcomes: daily level of stress and daily level of stress expected for the following day. In the sampling procedure, we used two chains, 5000 burn-in steps, and 10,000 iterations as described in previous publications^68^. As no reasonable effect estimation for the two investigated anti-stress interventions on daily stress levels in physicians was available prior to conducting this study, we chose the conservative approach and used non-informative priors. In the Bayesian analyses, a first-order autoregressive error structure (AR1) modeling the dependency between days was used. Bayesian models allow a probabilistic description of the data and can be used to derive the probability of reaching a specified clinically relevant effect. Here, a clinically relevant effect was defined as a stress reduction of at least 0.5 points on the 0 to 10 stress scale. As the individual burden to perform the intervention was low, we a priori defined participants with a probability of at least 70% for reaching the clinically relevant effect as responders. The one-sided cumulative probabilities of reaching effect sizes below zero and below 0.5 points were calculated. We want to point out that by providing the data on the individual level probabilities (Figs. 2C, D and 3C, D) as well as for the population level (Fig. 4), it is possible to infer the probability of achieving clinically meaningful effects for alternative effect sizes beyond the threshold defined as clinically relevant in this study. As no consensus on the clinically relevant effect as well as on the threshold for refining a responder is available, and the evaluation of which effect size as well as which probability of reaching is most likely different for everybody, this flexibility in the interpretation is one of the major advantages of the N-of-1 trial design.

An intention-to-treat analysis was performed. All available data points were included in the analyses, independent of the number of reported outcomes or if the trial was completed. The role of potential effect modifiers was exploratively analyzed in subgroup analyses. All statistical analyses were conducted using the R software package version 4.4.1^107^. Bayesian models were calculated using the brms-package. Figures were drawn with the ggplot2 package. Cronbachs’s alpha was calculated using the alpha-function from the psych package.

### Ethics

All participants provided informed consent. The trial was conducted in accordance with the Declaration of Helsinki and approved by the Ethics Committee of the Charité—Universitätsmedizin Berlin (approval number EA4/260/23). It is registered at ClinicalTrials.gov under trial ID NCT06368791 (first registered on April 11, 2024).

## Supplementary information

Supplements.

Checklist.

## Data Availability

The study setup, all information on the protocol of the conducted N-of-1 trials as well as all data collected as part of the N-of-1 trials are publicly available through the StudyU data repository at https://designer.studyu.health (mindfulness breathing: “ASIP-Studie: Anti-Stress Übung”, box breathing: “ASIP-Studie: Box-Atmung”). All data used to produce the results shown in this manuscript (including the data from the baseline and follow-up questionnaires collected through REDCap) are published at https://github.com/HIAlab/ASIP. Statistical code used to produce all statistical analyses and figures is available at https://github.com/HIAlab/ASIP.
