# Supplementary material for: Evaluation of two easy-to-implement digital breathing interventions in the context of daily stress levels in a series of N-of-1 trials: results from the Anti-Stress Intervention Among Physicians (ASIP) study
Source: NPJ Digit Med. 2026 Jan 10;9:135. doi: 10.1038/s41746-025-02317-3 (PMC12881409; doi:10.1038/s41746-025-02317-3)
Supplement: Supplementary file 1 — Supplements. [file 41746_2025_2317_MOESM1_ESM.pdf]

Supplementary Materials

Evaluation of two easy-to-implement digital breathing interventions in the context of daily stress levels in a series of N-of-1 trials: results from the Anti-Stress Intervention Among Physicians (ASIP) Study

Valentin Max Vetter, Tobias Kurth, Stefan Konigorski

Supplementary Figures

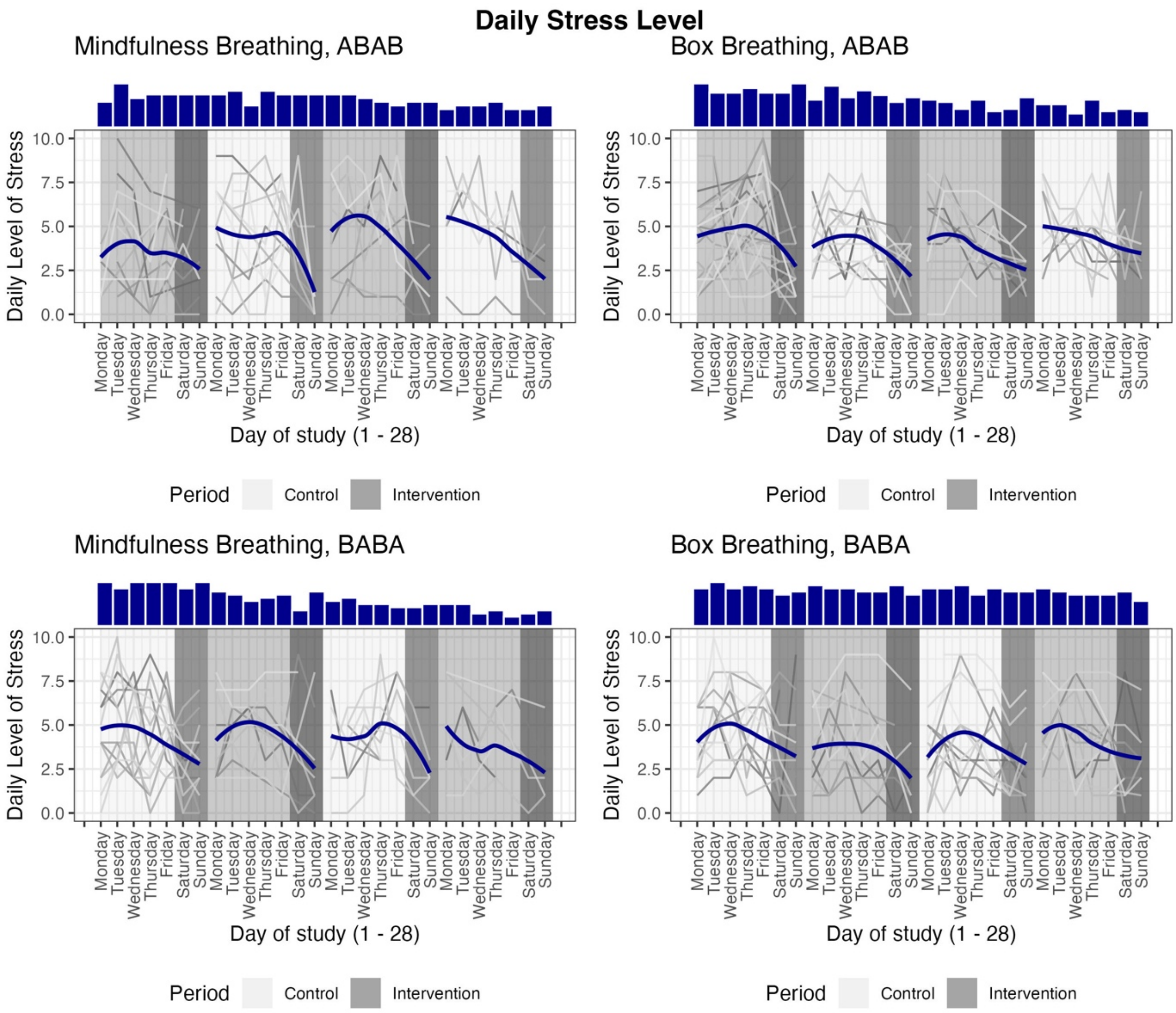

Supplementary Figure 1: *Individual line plots display the daily stress levels of each participant, stratified by their preferred intervention (Mindfulness Breathing or Box Breathing) and the randomly assigned trial sequence (A = intervention period, B: = control period). The blue line was fitted to all data points collected to allow interpretation of overall trends. The marginal histograms report the number of observations per day of study. To facilitate comparison, the data for each participant has been restructured to align on a common timeline. As a result, two consecutive data points may not correspond to consecutive days of data collection for each participant and the individual calendry day of study participation at one specific day on the x-axis differs between participants.*

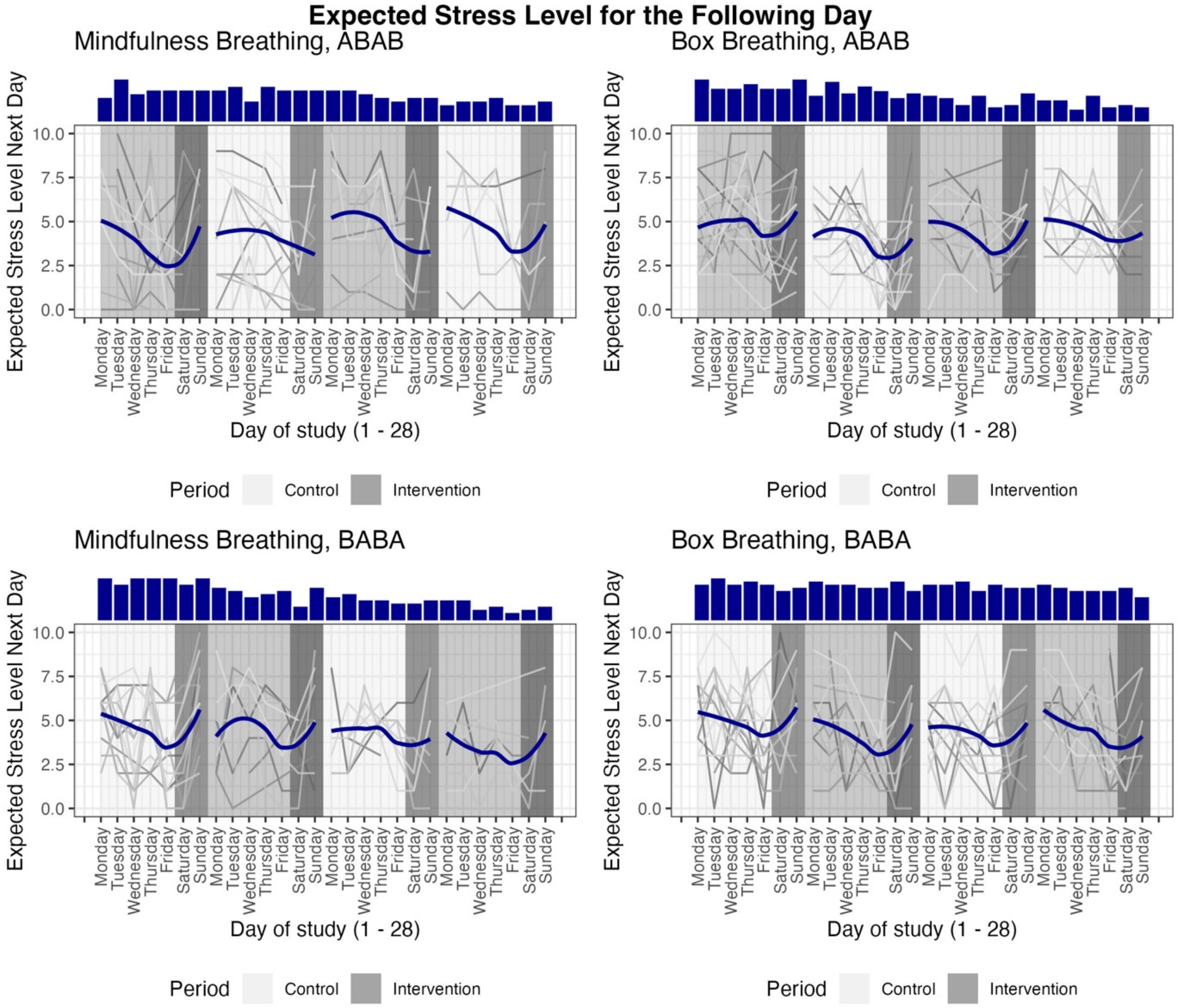

Supplementary Figure 2*: Individual line plots display the stress levels expected for the next day of each participant, stratified by their preferred intervention (Mindfulness Breathing or Box Breathing) and the randomly assigned trial sequence (A: intervention period, B: control period). The blue line was fitted to all data points collected to allow interpretation of overall trends. The marginal histograms report the number of observations per day of study. To facilitate comparison, the data for each participant has been restructured to align on a common timeline. As a result, two consecutive data points may not correspond to consecutive days of data collection for each participant and the individual calendry day of study participation at one specific day on the x-axis differs between participants.*

Supplementary Tables

Supplementary Table 1: *Individual level average intervention effect of performing Mindfulness Breathing on the daily stress level. Bayesian models using an autoregressive error structure (AR1) are used to estimate the mean posterior distribution and 95% credible interval. Additionally, the probability to reach a clinically relevant stress reduction of at least 0.5 points is given.*

**ID**	**Intervention**			**Control**			**Diff.**	**Estim.**	**l 95% CI**	**u 95% CI**	**p. clin. rel.**
	**Mean**	**SD**	**n**	**Mean**	**SD**	**n**					
P_1	3.9	1.66	10	5.92	2.14	13	-2.02	-1.37	-3.49	0.85	0.7920
P_5	3.57	1.99	7	4.62	2.26	8	-1.05	-0.25	-3.28	3.87	0.4943
P_11	5	1.77	8	5	2.58	7	0	-0.71	-4.02	2.16	0.5356
P_17	2	1.41	4	3.2	1.3	5	-1.2	-1.14	-3.33	0.86	0.7545
P_18	5.4	2.61	5	3.89	1.83	9	1.51	0.93	-2.62	4.08	0.1782
P_19			0			1					
P_20	5.44	2.7	9	6.62	2.13	8	-1.18	-0.46	-3.23	2.33	0.4863
P_22			1			7					
P_24			3			0					
P_25			0			7					
P_28	3.22	1.79	9	3.4	1.14	5	-0.18	0.56	-1.93	3.63	0.2262
P_29	0.92	1	12	0.62	0.65	13	0.3	0.30	-0.48	1.08	0.0227
P_30			1			7					
P_33	2.25	0.96	4	3	1	3	-0.75	-0.57	-2.52	2.41	0.6352
P_35	4.6	2.07	5	4.46	2.73	13	0.14	0.24	-2.94	3.46	0.3120
P_37			1			0					
P_38	3.85	2.27	13	5.07	2.73	14	-1.23	-1.08	-3.36	1.42	0.6975
P_39	2.25	1.89	4	2.25	3.3	4	0	-0.13	-4.36	4.10	0.4231
P_42	2.36	2.1	14	2.64	3.03	14	-0.29	-0.23	-2.71	2.34	0.4125
P_43	4.33	2.07	6	5.17	2.33	12	-0.83	-0.35	-2.90	2.22	0.4533
P_46	5.11	2.47	9	5.1	2.73	10	0.01	0.39	-2.62	3.70	0.2853
P_47	3.93	1.33	14	3.79	2.15	14	0.14	0.74	-1.17	2.77	0.1027
P_53	5.2	0.84	5	5.75	0.96	4	-0.55	-0.61	-2.38	0.99	0.5430
P_54			0			1					
P_55	2.79	3.36	14	2.86	2.88	14	-0.07	-1.07	-4.33	2.06	0.6397
P_58			1			0					
P_60			3			0					
P_63	6.33	2.35	9	5.43	1.81	7	0.9	0.65	-1.01	2.60	0.0814
P_64	2.5	2.17	10	3	1.68	13	-0.5	-0.63	-2.75	1.20	0.5378
P_69	4.5	2.43	6	4.5	3.67	6	0	0.03	-3.27	3.43	0.3519
P_70	3.64	2.5	14	4.5	2.14	14	-0.86	-0.91	-3.20	1.42	0.6391
P_71	5.88	1.64	8	5	1.93	8	0.88	1.10	-1.04	3.53	0.0633
P_73	7.17	0.75	6	4	1.41	5	3.17	3.12	1.70	4.63	0.0006

Note: SD = Standard Deviation; n = number of observations; Diff. = mean difference of observations between intervention and control periods; Estim. = mean posterior distribution; CI = credible interval; p. clin. rel. = probability of achieving clinically relevant effect.

Supplementary Table 2: *Individual level average intervention effect of performing the Box Breathing on the daily stress level. Bayesian models using an autoregressive error structure (AR1) are used to* estimate *the mean posterior distribution and 95% credible interval. Additionally, the probability to reach a clinically relevant stress reduction of at least 0.5 points is given.*

ID	Intervention			Control			Diff.	Estim.	l 95% CI	u 95% CI	p. clin. rel.
	Mean	SD	n	Mean	SD	n					
P_2	3.36	1.95	14	3.5	2.21	14	-0.14	-0.17	-2.23	1.82	0.3627
P_3	1.6	1.65	10	2.62	1.8	13	-1.02	-1.04	-2.38	0.34	0.8055
P_4	4.09	1.81	11	3.67	1.87	12	0.42	0.54	-1.48	2.59	0.1439
P_6	4	2.37	11	3.9	1.66	10	0.1	-0.17	-2.93	1.96	0.3507
P_7	5.08	1.93	13	3.25	1.6	12	1.83	1.50	-0.56	3.39	0.0284
P_8	5.12	1.96	8	2.71	0.76	7	2.41	2.45	0.49	4.47	0.0034
P_9	5.6	3.05	5	4.67	1.53	3	0.93	0.07	-6.42	5.04	0.3728
P_10			4			1					
P_12	2.43	1.81	7	4	2.19	6	-1.57	-2.18	-5.29	0.75	0.8831
P_13			2			0					
P_14	4	2	9	4.38	1.41	8	-0.38	-0.08	-2.11	2.44	0.3773
P_15			2			1					
P_16	2.56	1.51	9	5.67	2.8	6	-3.11	-2.97	-5.51	0.01	0.9546
P_21	4.62	2.45	8	3.14	2.19	7	1.48	1.68	-1.26	4.73	0.065
P_23			2			0					
P_26	4.45	0.69	11	4	1.86	12	0.45	0.57	-1.04	2.19	0.0894
P_27			3			0					
P_31	6	1.41	2	6.17	1.17	6	-0.17	0.12	-2.43	2.61	0.2911
P_32	4.14	1.68	7	4.6	2.07	5	-0.46	-0.30	-3.44	1.91	0.3815
P_34			6			1					
P_36	2.27	0.9	11	3.67	0.98	12	-1.39	-1.34	-2.20	-0.61	0.9854
P_40	4	1.07	8	5	0.93	8	-1	-1.00	-2.28	0.29	0.7938
P_41	2.31	1.32	13	3.31	2.87	13	-1	-0.39	-2.71	2.37	0.487
P_44			0			1					
P_45			3			0					
P_48	3.54	2.18	13	2.45	1.97	11	1.08	0.82	-1.52	2.99	0.119
P_49			3			0					
P_50	4.08	2.81	12	5.36	2.5	14	-1.27	-1.29	-3.74	0.88	0.7644
P_51			4			1					
P_52	3	1.47	14	3.79	1.58	14	-0.79	-0.74	-2.19	0.77	0.633
P_56	2.8	2.28	5	4.5	2.66	6	-1.7	-1.74	-5.88	1.71	0.7616
P_57			0			4					
P_59	3.38	0.92	8	4.1	0.88	10	-0.72	-0.60	-1.67	0.56	0.586
P_61	2.57	1.27	7	2.36	1.12	11	0.21	0.73	-0.75	2.26	0.0489
P_62	4.62	1.61	13	4.6	2.41	10	0.02	-0.16	-2.40	2.07	0.369
P_65	6.08	1.38	12	5.31	1.6	13	0.78	0.71	-0.82	2.15	0.055
P_66	4	1.31	8	3.8	0.92	10	0.2	0.25	-1.11	1.66	0.1243
P_67	1.57	0.79	7	1	1	7	0.57	0.50	-1.06	1.65	0.0705
P_68	5.75	1.82	12	5.5	1.61	14	0.25	0.24	-1.16	1.68	0.1421
P_72	5	2.06	9	4.89	0.78	9	0.11	0.07	-1.80	1.87	0.2494
P_74	3.14	1.83	14	3.77	1.48	13	-0.63	-0.62	-2.02	0.71	0.5656
P_75	7.33	1.32	9	6.92	2.19	12	0.42	0.33	-1.91	2.51	0.2125
P_76	2.82	2.75	11	2.6	1.67	5	0.22	-1.03	-4.52	2.49	0.6169

Note: SD = Standard Deviation; n = number of observations; Diff. = mean difference of observations between intervention and control periods; Estim. = mean posterior distribution; CI = credible interval; p. clin. rel. = probability of achieving clinically relevant effect.

Supplementary Table 3: *Individual-level average intervention effect of performing the Mindfulness Breathing on the stress level expected for the next day. Bayesian models using an autoregressive error structure (AR1) are used to* estimate *the mean posterior distribution and 95% credible interval. Additionally, the probability to reach a clinically relevant stress reduction of at least 0.5 points is given.*

ID	Intervention			Control			Diff.	Estim.	l 95% CI	u 95% CI	p. clin. rel.
	Mean	SD	n	Mean	SD	n			Mean	SD	n
P_1	3.9	1.66	10	5.92	2.14	13	-2.02	-1.18	-3.50	1.27	0.7283
P_5	3.57	1.99	7	4.62	2.26	8	-1.05	0.00	-3.15	3.61	0.3944
P_11	5	1.77	8	5	2.58	7	0	-0.31	-3.54	2.13	0.3944
P_17	2	1.41	4	3.2	1.3	5	-1.2	-1.18	-3.06	0.60	0.8039
P_18	5.4	2.61	5	3.89	1.83	9	1.51	-0.39	-2.62	1.91	0.4554
P_19			0			1					
P_20	5.44	2.7	9	6.62	2.13	8	-1.18	-0.86	-2.70	0.78	0.6697
P_22			1			7					
P_24			3			0					
P_25			0			7					
P_28	3.22	1.79	9	3.4	1.14	5	-0.18	-0.01	-2.30	2.29	0.3259
P_29	0.92	1	12	0.62	0.65	13	0.3	0.20	-1.15	1.47	0.1384
P_30			1			7					
P_33	2.25	0.96	4	3	1	3	-0.75	0.71	-3.00	4.03	0.2133
P_35	4.6	2.07	5	4.46	2.73	13	0.14	0.29	-1.98	2.58	0.2404
P_37			1			0					
P_38	3.85	2.27	13	5.07	2.73	14	-1.23	-0.16	-2.96	2.72	0.4024
P_39	2.25	1.89	4	2.25	3.3	4	0	0.25	-1.44	1.78	0.1438
P_42	2.36	2.1	14	2.64	3.03	14	-0.29	-0.31	-2.84	3.21	0.4864
P_43	4.33	2.07	6	5.17	2.33	12	-0.83	0.50	-1.67	2.92	0.1761
P_46	5.11	2.47	9	5.1	2.73	10	0.01	-0.77	-2.71	1.21	0.6195
P_47	3.93	1.33	14	3.79	2.15	14	0.14	-0.57	-2.39	1.24	0.5338
P_53	5.2	0.84	5	5.75	0.96	4	-0.55	0.99	-0.99	2.62	0.059
P_54			0			1					
P_55	2.79	3.36	14	2.86	2.88	14	-0.07	2.29	-0.80	5.98	0.0386
P_58			1			0					
P_60			3			0					
P_63	6.33	2.35	9	5.43	1.81	7	0.9	0.25	-1.75	1.97	0.174
P_64	2.5	2.17	10	3	1.68	13	-0.5	-0.67	-2.63	1.35	0.5732
P_69	4.5	2.43	6	4.5	3.67	6	0	-1.67	-7.94	2.06	0.6651
P_70	3.64	2.5	14	4.5	2.14	14	-0.86	-0.64	-3.29	2.28	0.5542
P_71	5.88	1.64	8	5	1.93	8	0.88	0.10	-2.27	2.42	0.2829
P_73	7.17	0.75	6	4	1.41	5	3.17	1.75	-0.51	3.87	0.0255

Note: SD = Standard Deviation; n = number of observations; Diff. = mean difference of observations between intervention and control periods; Estim. = mean posterior distribution; CI = credible interval; p. clin. rel. = probability of achieving clinically relevant effect.

Supplementary Table 4: *Individual-level average intervention effect of performing the Box Breathing on the stress level expected for the next day. Bayesian models using an autoregressive error structure (AR1) are used to estimate the mean posterior distribution and 95% credible interval. Additionally, the probability to reach a clinically relevant stress reduction of at least 0.5 points is given.*

**ID**	**Intervention**			**Control**			**Diff.**	**Estim.**	**l 95% CI**	**u 95% CI**	**p. clin. rel.**
	**Mean**	**SD**	**n**	**Mean**	**SD**	**n**					
P_2	3.36	1.95	14	3.5	2.21	14	-0.14	0.34	-1.93	2.87	0.2297
P_3	1.6	1.65	10	2.62	1.8	13	-1.02	-0.49	-2.12	1.33	0.5094
P_4	4.09	1.81	11	3.67	1.87	12	0.42	0.43	-1.69	3.10	0.2163
P_6	4	2.37	11	3.9	1.66	10	0.1	-0.22	-2.40	1.96	0.3887
P_7	5.08	1.93	13	3.25	1.6	12	1.83	-0.06	-2.47	1.98	0.3259
P_8	5.12	1.96	8	2.71	0.76	7	2.41	1.52	-0.44	3.47	0.0229
P_9	5.6	3.05	5	4.67	1.53	3	0.93	-0.02	-2.71	3.76	0.3845
P_10			4			1					
P_12	2.43	1.81	7	4	2.19	6	-1.57	-0.54	-2.91	1.96	0.5201
P_13			2			0					
P_14	4	2	9	4.38	1.41	8	-0.38	0.05	-2.82	2.76	0.3246
P_15			2			1					
P_16	2.56	1.51	9	5.67	2.8	6	-3.11	-1.56	-4.09	1.08	0.8111
P_21	4.62	2.45	8	3.14	2.19	7	1.48	0.56	-2.54	3.44	0.2278
P_23			2			0					
P_26	4.45	0.69	11	4	1.86	12	0.45	0.24	-1.19	1.74	0.1493
P_27			3			0					
P_31	6	1.41	2	6.17	1.17	6	-0.17	-0.33	-3.85	3.07	0.4434
P_32	4.14	1.68	7	4.6	2.07	5	-0.46	0.15	-1.78	1.91	0.2143
P_34			6			1					
P_36	2.27	0.9	11	3.67	0.98	12	-1.39	-0.39	-1.32	0.56	0.3982
P_40	4	1.07	8	5	0.93	8	-1	-0.52	-1.43	0.41	0.5174
P_41	2.31	1.32	13	3.31	2.87	13	-1	-1.23	-3.34	1.44	0.7708
P_44			0			1					
P_45			3			0					
P_48	3.54	2.18	13	2.45	1.97	11	1.08	-1.16	-3.16	0.88	0.7466
P_49			3			0					
P_50	4.08	2.81	12	5.36	2.5	14	-1.27	0.49	-1.80	3.06	0.2019
P_51			4			1					
P_52	3	1.47	14	3.79	1.58	14	-0.79	-0.33	-2.05	1.48	0.4208
P_56	2.8	2.28	5	4.5	2.66	6	-1.7	0.41	-3.24	4.56	0.322
P_57			0			4					
P_59	3.38	0.92	8	4.1	0.88	10	-0.72	-0.78	-2.17	0.49	0.67
P_61	2.57	1.27	7	2.36	1.12	11	0.21	0.03	-0.98	1.04	0.1297
P_62	4.62	1.61	13	4.6	2.41	10	0.02	1.14	-0.77	3.43	0.0435
P_65	6.08	1.38	12	5.31	1.6	13	0.78	0.27	-0.97	1.41	0.0902
P_66	4	1.31	8	3.8	0.92	10	0.2	1.01	-0.53	2.43	0.0276
P_67	1.57	0.79	7	1	1	7	0.57	0.22	-1.10	1.47	0.1252
P_68	5.75	1.82	12	5.5	1.61	14	0.25	1.02	-0.54	2.61	0.0282
P_72	5	2.06	9	4.89	0.78	9	0.11	2.03	0.86	3.20	4e-04
P_74	3.14	1.83	14	3.77	1.48	13	-0.63	-0.42	-1.64	0.74	0.4347
P_75	7.33	1.32	9	6.92	2.19	12	0.42	-0.19	-2.02	1.60	0.3517
P_76	2.82	2.75	11	2.6	1.67	5	0.22	-0.24	-3.50	2.96	0.428

Note: SD = Standard Deviation; n = number of observations; Diff. = mean difference of observations between intervention and control periods; Estim. = mean posterior distribution; CI = credible interval; p. clin. rel. = probability of achieving clinically relevant effect.

Supplementary Table 5: *Sex-stratified mean posterior, 95% credible interval and posterior probability to reach a clinically relevant stress reduction of at least 0.5 points in 60 women and 17 men participating in the ASIP study. Additionally, sex-stratified mean, standard deviation, number of documented PROs and mean difference in outcome variables between intervention and control periods are shown.*

Outcome	Anti-Stress Exercise	Intervention Period			Control Period			Diff.	Estim.	Lower 95% CI	Upper 95% CI	p. clin. rel.
		Mean	SD	n	Mean	SD	n				Mean	SD
**Women**												
Daily Stress	Mindfulness Breathing	3.94	2.54	199	3.89	2.6	214	0.05	-0.03	-0.50	0.45	0.025
	Box Breathing	4.11	2.22	241	4.3	1.99	219	-0.19	-0.09	-0.52	0.34	0.0298
Stress Next Day	Mindfulness Breathing	4.07	2.75	199	4.15	2.7	214	-0.09	-0.17	-0.61	0.27	0.0724
	Box Breathing	4.54	2.22	241	4.54	1.97	219	0	0.14	-0.25	0.54	0.0005
**Men**												
Daily Stress	Mindfulness Breathing	4.06	1.77	16	5.38	2.17	32	-1.31	-1.25	-4.32	2.40	0.7493
	Box Breathing	3.42	2.25	90	3.32	2.15	95	0.11	0.11	-0.69	0.95	0.0666
Stress Next Day	Mindfulness Breathing	4.31	1.96	16	5.22	1.95	32	-0.91	-0.57	-6.77	3.39	0.4837
	Box Breathing	4.17	2.37	90	3.91	2.41	95	0.26	0.24	-0.44	0.96	0.0179

Note: SD = Standard Deviation; n = number of observations; Diff. = Difference between average stress levels in intervention and control period; Estim. = mean posterior distribution; CI = credible interval; p. clin. rel. = probability of achieving clinically relevant effect.

Supplementary Table 6: *Mean posterior, 95% credible interval and posterior probability to reach a clinically relevant stress reduction of at least 0.5 points stratified by baseline stress levels assessed by Cohen's PSS. A median split was performed to compare participants below the 50th percentile with participants above the 50th percentile. Additionally, mean, standard deviation, number of documented PROs and mean difference in outcome variables between intervention and control periods are shown for the respective subgroups.*

Outcome	Anti-Stress Exercise	Intervention Period			Control Period			Diff.	Estim.	Lower 95% CI	Upper 95% CI	% clin. rel.
		Mean	SD	n	Mean	SD	n					
**Low Chronic Stress at Baseline**												
Daily Stress	Mindfulness Breathing	3.72	2.28	95	3.79	2.64	120	-0.08	-0.04	-0.73	0.65	0.084
	Box Breathing	3.30	2	174	3.43	2.04	152	-0.14	-0.32	-0.97	0.31	0.269
Stress Next Day	Mindfulness Breathing	3.66	2.57	95	3.85	2.64	120	-0.19	-0.05	-0.67	0.58	0.077
	Box Breathing	3.69	2.06	174	3.78	1.84	152	-0.09	-0.17	-0.65	0.36	0.0877
**High Chronic Stress at Baseline**												
Daily Stress	Mindfulness Breathing	4.13	2.64	120	4.37	2.52	126	-0.23	-0.25	-0.92	0.44	0.229
	Box Breathing	4.58	2.29	166	4.52	1.97	170	0.05	0.13	-0.35	0.64	0.0047
Stress Next Day	Mindfulness Breathing	4.42	2.76	120	4.71	2.57	126	-0.3	-0.29	-0.96	0.38	0.265
	Box Breathing	5.21	2.22	166	4.85	2.25	170	0.36	0.38	-0.06	0.83	0.0001

Note: SD = Standard Deviation; n = number of observations; Diff. = Difference between average stress levels in intervention and control period; Estim. = mean posterior distribution; CI = credible interval; p. clin. rel. = probability of achieving clinically relevant effect, PSS = Perceived Stress Scale.

Supplementary Table 7: *Average intervention effect on the daily stress level and the level of stress expected for the following day of to the availability of an anti-stress intervention among the participants that chose the respective intervention. The dataset analyzed here contains all PROs documented by participants that chose to evaluate Mindfulness Breathing (intervention 1) or Box Breathing (intervention 2). Mean posterior, 95% credible interval and probability to reach a reduction of at least 0.5 points on the respective stress scales were estimated by Bayesian multilevel models. Mean, standard deviation and number of documented PROs in the analyzed dataset are displayed.*

Data and Outcome	**Intervention Period**			**Control Period**			**Diff.**	**Estim.**	**Lower 95% CI**	**Upper 95% CI**	**p. clin. rel.**
	**Mean**	**SD**	**n**	**Mean**	**SD**	**n**					
Across interventions (1 + 2), Daily Stress	3.93	2.34	555	4.04	2.31	568	-0.11	-0.10	-0.38	0.17	0.0026
Across interventions (1 + 2), Stress Next Day	4.30	2.45	555	4.32	2.36	568	-0.02	0.00	-0.24	0.24	<0.0001

Note: SD = Standard Deviation; n = number of observations; Diff. = Difference between average stress levels in intervention and control period; Estim. = mean posterior distribution; CI = credible interval; p. clin. rel. = probability of achieving clinically relevant effect.
