# Supplementary material for: Evaluation of two easy-to-implement digital breathing interventions in the context of daily stress levels in a series of N-of-1 trials: results from the Anti-Stress Intervention Among Physicians (ASIP) study
Source: NPJ Digit Med. 2026 Jan 10;9:135. doi: 10.1038/s41746-025-02317-3 (PMC12881409; doi:10.1038/s41746-025-02317-3)
Supplement: Supplementary file 2 — Checklist. [file 41746_2025_2317_MOESM2_ESM.pdf]

Table 1 | CENT 2015 checklist*; CONSORT 2010 checklist items with modifications or additions for individual or series of N-of-1 trials; empty items in the CENT 2015 column indicate no modification from the CONSORT 2010 item

Section/Topic	CONSORT 2010 No	CONSORT 2010 Item	CENT 2015 No	CENT 2015 Item	Page no.
**Title and abstract**					
	1a	Identification as a randomised trial in the title	1a	Identify as an "N-of-1 trial" in the title<br>*For series:* Identify as "a series of N-of-1 trials" in the title	1
	1b	Structured summary of trial design, methods, results, and conclusions (for specific guidance see CONSORT for abstracts)	1b	For specific guidance, see CENT guidance for abstracts (table 2)	2
**Introduction**					
Background and objectives	2a	Scientific background and explanation of rationale	2a.1		3
			2a.2	Rationale for using N-of-1 approach	3
	2b	Specific objectives or hypotheses	2b		4
**Methods**					
Trial design	3a	Description of trial design (such as parallel, factorial) including allocation ratio	3a	Describe trial design, planned number of periods, and duration of each period (including run-in and wash out, if applicable)<br>*In addition for series:* Whether and how the design was individualized to each participant, and explain the series design	13-17
	3b	Important changes to methods after trial start (such as eligibility criteria), with reasons	3b		n.a.
Participant(s)	4a	Eligibility criteria for participants	4a†	Diagnosis or disorder, diagnostic criteria, comorbid conditions, and concurrent therapies.<br>*For series:* Same as CONSORT item 4a	13, 14
	4b	Settings and locations where the data were collected	4b†		14
			4c	Whether the trial(s) represents a Research Methods & Reporting study and if so, whether institutional ethics approval was obtained	14
Interventions	5	The interventions for each group with sufficient details to allow replication, including how and when they were actually administered	5	The interventions for each period with sufficient details to allow replication, including how and when they were actually administered	14
Outcomes	6a	Completely defined pre-specified primary and secondary outcome measures, including how and when they were assessed	6a.1		15
			6a.2	Description and measurement properties (validity and reliability) of outcome assessment tools	15
	6b	Any changes to trial outcomes after the trial commenced, with reasons	6b		n.a.
Sample size	7a	How sample size was determined	7a		22
	7b	When applicable, explanation of any interim analyses and stopping guidelines	7b		n.a.
Randomisation:					
Sequence generation	8a	Method used to generate the random allocation sequence	8a	Whether the order of treatment periods was randomised, with rationale, and method used to generate allocation sequence	15
	8b	Type of randomisation; details of any restriction (such as blocking and block size)	8b	When applicable, type of randomisation; details of any restrictions (such as pairs, blocking)	15
			8c	Full, intended sequence of periods	15
Allocation concealment mechanism	9	Mechanism used to implement the random allocation sequence (such as sequentially numbered containers), describing any steps taken to conceal the sequence until interventions were assigned	9		15
Implementation	10	Who generated the random allocation sequence, who enrolled participants, and who assigned participants to interventions	10		15
Blinding	11a	If done, who was blinded after assignment to interventions (for example, participants, care providers, those assessing outcomes) and how	11a		n.a.
	11b	If relevant, description of the similarity of interventions	11b		14
Statistical methods	12a	Statistical methods used to compare groups for primary and secondary outcomes	12a	Methods used to summarize data and compare interventions for primary and secondary outcomes	17
	12b	Methods for additional analyses, such as subgroup analyses and adjusted analyses	12b	*For series:* If done, methods of quantitative synthesis of individual trial data, including subgroup analyses, adjusted analyses, and how heterogeneity between participants was assessed *(for specific guidance on reporting syntheses of multiple trials, please consult the PRISMA Statement)*	14, 17
			12c	Statistical methods used to account for carryover effect, period effects, and intra-subject correlation	17

(Continued)

Table 1 | (Continued) CENT 2015 checklist*; CONSORT 2010 checklist items with modifications or additions for individual or series of N-of-1 trials; empty items in the CENT 2015 column indicate no modification from the CONSORT 2010 item

	CONSORT 2010		CENT 2015		Page no.
Section/Topic	No	Item	No	Item	
**Results**					
Participant flow (a diagram is strongly recommended)	13a	For each group, the numbers of participants who were randomly assigned, received intended treatment, and were analysed for the primary outcome	13a.1	Number and sequence of periods completed, and any changes from original plan with reasons	4, Fig.1
			13a.2	*For series:* The number of participants who were enrolled, assigned to interventions, and analysed for the primary outcome	4, Fig.1
	13b	For each group, losses and exclusions after randomisation, together with reasons	13c	*For series:* Losses or exclusions of participants after treatment assignment, with reasons, and period in which this occurred, if applicable	4, Fig.1
Recruitment	14a	Dates defining the periods of recruitment and follow-up	14a†		14
	14b	Why the trial ended or was stopped	14b	Whether any periods were stopped early and/or whether trial was stopped early, with reason(s).	n.a.
Baseline data	15	A table showing baseline demographic and clinical characteristics for each group	15†		Table 1
Numbers analysed	16	For each group, number of participants (denominator) included in each analysis and whether the analysis was by original assigned groups	16	For each intervention, number of periods analysed. *In addition for series:* If quantitative synthesis was performed, number of trials for which data were synthesized	Fig.1, Table 1
Outcomes and estimation	17a	For each primary and secondary outcome, results for each group, and the estimated effect size and its precision (such as 95% confidence interval)	17a.1	For each primary and secondary outcome, results for each period; an accompanying figure displaying the trial data is recommended.	Fig.2 -4, Suppl. Table 1-4, Suppl. Fig. 1-2
			17a.2	For each primary and secondary outcome, the estimated effect size and its precision (such as 95% confidence interval) *In addition for series:* If quantitative synthesis was performed, group estimates of effect and precision for each primary and secondary outcome	Table 2, Suppl. Table 1-7
	17b	For binary outcomes, presentation of both absolute and relative effect sizes is recommended	17b		n.a.
Ancillary analyses	18	Results of any other analyses performed, including subgroup analyses and adjusted analyses, distinguishing pre-specified from exploratory	18	Results of any other analyses performed, including assessment of carryover effects, period effects, intra-subject correlation *In addition for series:* If done, results of subgroup or sensitivity analyses	Suppl. Table 5-7
Harms	19	All important harms or unintended effects in each group (for specific guidance see CONSORT for harms)	19	All harms or unintended effects for each intervention. *(for specific guidance see CONSORT for harms)*	11
**Discussion**					
Limitations	20	Trial limitations, addressing sources of potential bias, imprecision, and, if relevant, multiplicity of analyses	20		12
Generalisability	21	Generalisability (external validity, applicability) of the trial findings	21		13
Interpretation	22	Interpretation consistent with results, balancing benefits and harms, and considering other relevant evidence	22		12-13
**Other information**					
Registration	23	Registration number and name of trial registry	23		14
Protocol	24	Where the full trial protocol can be accessed, if available	24		13
Funding	25	Sources of funding and other support (such as supply of drugs), role of funders	25		18

*It is strongly recommended that this checklist be read in conjunction with the CENT 2015 Explanation and Elaboration[24] for important clarification on the items. The copyright for CENT (including checklist) is held by the CENT Group and is distributed under a Creative Commons Attribution (CC-BY 4.0) license.
†Caution should be taken when reporting potentially identifying information pertaining to CENT items 4a, 4b, 14a, and 15.
